# Comparison and Combination of Dual-Energy- and Iterative-Based Metal Artefact Reduction on Hip Prosthesis and Dental Implants

**DOI:** 10.1371/journal.pone.0143584

**Published:** 2015-11-24

**Authors:** Malte N. Bongers, Christoph Schabel, Christoph Thomas, Rainer Raupach, Mike Notohamiprodjo, Konstantin Nikolaou, Fabian Bamberg

**Affiliations:** 1 Department of Diagnostic and Interventional Radiology, University Hospital of Tübingen, Hoppe-Seyler-Strasse 3, 72076, Tübingen, Germany; 2 Department of Diagnostic and Interventional Radiology, University Hospital of Düsseldorf, Moorenstrasse 5, 40225, Düsseldorf, Germany; 3 Siemens AG, Healthcare Sector, Siemensstraße 1, 91301, Forchheim, Germany; Chongqing University, CHINA

## Abstract

**Purpose:**

To compare and combine dual-energy based and iterative metal artefact reduction on hip prosthesis and dental implants in CT.

**Material and Methods:**

A total of 46 patients (women:50%,mean age:63±15years) with dental implants or hip prostheses (n = 30/20) were included and examined with a second-generation Dual Source Scanner. 120kV equivalent mixed-images were derived from reconstructions of the 100/Sn140kV source images using no metal artefact reduction (NOMAR) and iterative metal artefact reduction (IMAR). We then generated monoenergetic extrapolations at 130keV from source images without IMAR (DEMAR) or from source images with IMAR, (IMAR+DEMAR). The degree of metal artefact was quantified for NOMAR, IMAR, DEMAR and IMAR+DEMAR using a Fourier-based method and subjectively rated on a five point Likert scale by two independent readers.

**Results:**

In subjects with hip prosthesis, DEMAR and IMAR resulted in significantly reduced artefacts compared to standard reconstructions (33% vs. 56%; for DEMAR and IMAR; respectively, p<0.005), but the degree of artefact reduction was significantly higher for IMAR (all p<0.005). In contrast, in subjects with dental implants only IMAR showed a significant reduction of artefacts whereas DEMAR did not (71%, vs. 8% p<0.01 and p = 0.1; respectively). Furthermore, the combination of IMAR with DEMAR resulted in additionally reduced artefacts (Hip prosthesis: 47%, dental implants 18%; both p<0.0001).

**Conclusion:**

IMAR allows for significantly higher reduction of metal artefacts caused by hip prostheses and dental implants, compared to a dual energy based method. The combination of DE-source images with IMAR and subsequent monoenergetic extrapolation provides an incremental benefit compared to both single methods.

## Introduction

Since the beginning of the development of computed tomography (CT), metallic implants, such as dental hardware, joint prostheses and osteosynthetic material have resulted in substantial artefacts with subsequent limited ability to evaluate adjacent anatomic structures [1]. This diagnostic challenge is particularly relevant as the demographic development is associated with an increasing prevalence of joint replacements and other implants [2]. However, in patients with metallic implants it is desirable to enable sufficient evaluation of the prosthesis itself, the interface between implant and bone and the surrounding soft tissue, regarding clinical questions such as fractures, implant loosening, hematoma, inflammation and malignancies [3].

Typical streaking artefacts from metallic implants occur predominantly due to two effects. First, photon starvation leads to an excessive increase of image noise, due to maximal attenuation by the metal implant and consecutive lack of photons reaching the detector. Second, beam hardening provokes insufficient soft tissue contrast in terms of dark bands [4].

Given the widespread application of CT, substantial efforts have been made to develop tools in order to reduce artefacts from metallic implants. High tube voltages of up to 140 kV result in reduction of beam hardening, monoenergetic images from dual-energy computed tomography (DECT) with virtual energies from 130–150 keV provide significant reduction of artefacts from metallic implants [5, 6]. Also, very early studies, using new iterative metal artefact reduction algorithms, show promising results in ex-vivo settings and initial human case series [7–9].

A new iterative software algorithm to reduce metal artefacts (IMAR, Siemens Healthcare, Germany) has been introduced, iteratively combining normalized sinogram interpolation with a frequency-split technique. The IMAR approach is based on the volumetric dataset and performs the forward projection step accounting for the geometry of the data acquisition during scanning, including exact spiral path and cone beam effects.

Furthermore, reducing metal artefacts in DECT-source images with subsequent monoenergetic extrapolation may theoretically have additional effects. However, alteration of the DECT-source images at different tube currents may also render monoenergetic extrapolation impossible. To our knowledge, no previous study has assessed this promising combination of metal artefact reduction techniques.

Therefore, the aim of this study was to a) compare and b) combine dual-energy based and iterative metal artefact reduction on hip prosthesis and dental implants in CT. Our hypothesis was that IMAR provides significantly higher reduction in metal artefacts compared to DEMAR, due to the frequency-split approach of the algorithm and that the combination of both techniques leads to a further reduction of metal artefacts.

## Material and Methods

### Patient population

The ethics committee of the University Hospital of Tübingen approved this retrospective study with a waiver of the need for informed consent. Analysis of patient study was performed in an anonymized and de-identified fashion. The study included consecutive oncological patients scheduled for standard whole body CT follow-up examinations. Imaging in all patients was indicated on a clinical basis and no CT examination was performed for study purpose only. Inclusion criteria were clinical indication for CT including the craniofacial area and/ or pelvis, as well as the presence of hip prosthesis or metallic dental implants. Patients under the age of 18 were excluded from this study. Patient Demographics and Imaging Parameters of the subjects are summarized in Table 1.

**Table 1:** Patient Demographics and Imaging Parameters of the 46 subjects included in the analysis. Data is given ± standard deviation.

Parameter	Value
**Gender**	
** Women**	23 (50%)
** Men**	23 (50%)
**Mean age** [y]	63.5 ± 15 (Range: 29–98)
**No. of hip prosthesis**	20
** No. of unilateral hip prostheses**	16
** No. of bilateral hip prostheses**	2
**No. of dental implants**	30
**Dose-length product** [mGy · cm]	809 ± 258
**Volume CT dose index** [mGy]	11.23 ± 2.99

### CT protocol and image reconstruction

All CT scans were performed using a second-generation DECT scanner (SOMATOM Definition Flash, Siemens Healthcare, Germany). Standard dual-energy protocol of the manufacturer with tube voltage combination of 100/Sn140 kV and an additional 0.4 mm tin filter was applied. Pitch was always set to 0.6 and collimation was 64 x 0.6 mm. Automated dose calculation was used, guaranteeing optimized dose for the whole examination. Depending on the patient’s weight and kidney function, 90 to 120 ml of contrast medium (400mg Iomeprol/ml, Imeron 400, Bracco, Konstanz, Germany) and 40 ml of saline chaser were injected through an antecubital vein catheter at a flow rate of about 2.2 ml/s using a dual-syringe injector (CT Stellant, Medrad, Indianola, Pennsylvania, USA). All images were reconstructed using a medium-soft reconstruction kernel (Q30f) with a slice thickness of 1.0 mm and an increment of 1.0 mm.

DECT based monoenergetic reconstructions (Monoenregetic, Siemens Healthcare, Germany) were calculated based on DE source images with and without using IMAR at 130 keV (DEMAR) as proposed by Zhou et al. [10]. The used algorithm overcomes previous limitations concerning image noise by using a frequency split technique, combining high contrast from low energy images with low noise levels from intermediate energy images [11].

IMAR combines two previously introduced MAR algorithms, i.e., normalized metal artefact reduction (NMAR) [12] and frequency-split metal artefact reduction (FSMAR) [13]. The aim of NMAR is to avoid the introduction of new artefacts tangentially to high contrast objects, which is often observed with other sinogram inpainting methods. The aim of FSMAR is to preserve both the natural image impression and the valid edge information of the uncorrected image, which is often affected by pure sinogram inpainting methods, especially in the vicinity of metal implants. IMAR repeatedly performs the normalized sinogram interpolation and frequency-split operations, using the result of each iteration as input for the next iteration. This effectively reduces the remaining artefacts of the prior image and consequently improves the quality of NMAR in each iteration. The algorithm is based on three to six reconstruction cycles based on the anticipated density of the metal implant.

Four different image sets were reconstructed from each examination as following:

1. **NOMAR**: No metal artefact reduction, DECT source images → Standard 120 kV-equivalent mixed images
2. **IMAR:** Iterative metal artefact reduction, DECT source images with IMAR → Standard 120 kV-equivalent mixed images
3. **DEMAR:** Dual Energy artefact reduction, DECT source images without metal artefact reduction → Monoenergetic images at 130 keV
4. **IMAR+DEMAR:** Combined iterative and dual energy metal artefact reduction: DECT source images with IMAR → Monoenergetic images at 130 keV

### Qualitative image analysis

**Fig 1:**
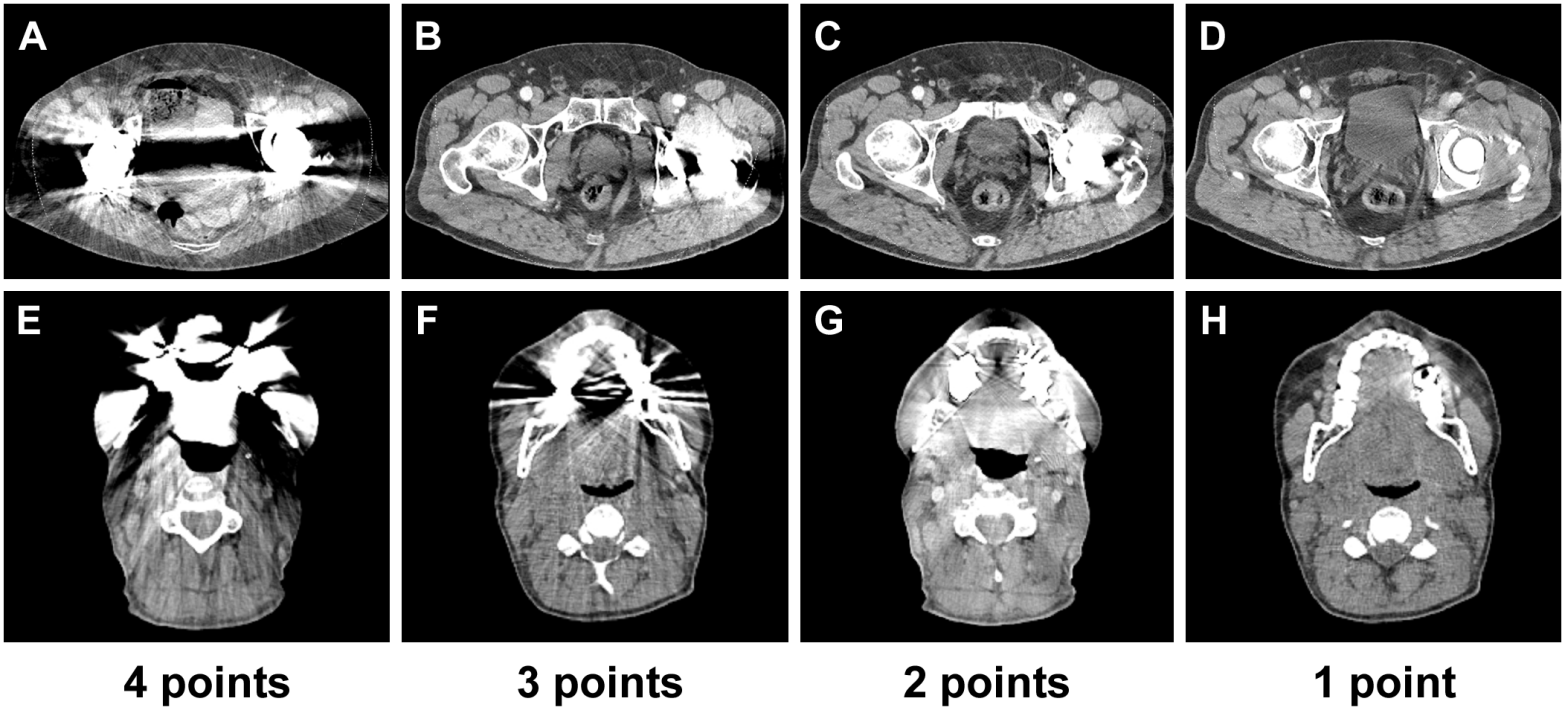
Qualitative image analysis is based on 5 point Likert scales taking into account the degree of artefact as well as diagnostic impact on adjacent and distant tissue for both hip prosthesis (A to D) and dental implants (E to H). A and E) massive artefacts (4 points), B and F) pronounced streaks (3 points), C and G) intermediate streaks (2 points), D and H) minimal streaks (1 point).

Subjective image analysis was performed in blinded fashion to the reconstruction method by two independent radiologists with three and four years of experience in body CT (M.N.B, C.S.). All reconstructions were primarily displayed in soft tissue window settings (Center 50 HU, Width 350 HU), but readers were allowed to adjust levels as desired. The applied 5 point Likert scales were defined as follows (Fig 1):

Artefacts are scored from 0 to 4, whereas 0 indicates the absence of artefacts, 1 indicates minimal streaks, 2 represents mild streaks, 3 indicates moderate streaks and 4 represents severe artefacts.

Diagnostic impact of artefacts was assessed as follows: (1) Adjacent tissue: Impact on surrounding tissue directly adjacent to the implant important; and (2) Distant tissue: Remote tissue on the same image slice but pertaining to diagnostically relevant anatomic structures (i.e. iliacal and inguinal lymph node levels or Pouch of Douglas).

Effect of metal artefacts was scored with 0 in fully diagnostic examinations, with 1 in diagnostic examinations without impairment from artefacts, with 2 in diagnostic examinations with little impairment from artefacts, with 3 in examinations with relevant impairment from artefacts and with 4 in non-diagnostic images.

### Quantitative image analysis

**Fig 2:**
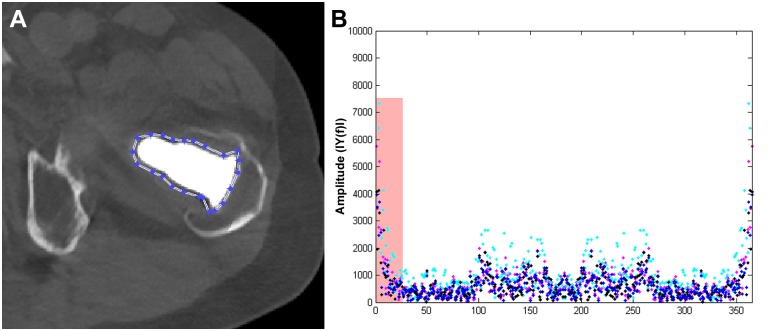
Applied method for quantitative image analysis with A) polygon placement around metallic implant to extract circular pixel information and B) results of discrete Fourier transform. Analysing amplitudes of low frequencies (red box) permits information on the degree of metal artefacts.

Artefact measurements in NOMAR, DEMAR, IMAR and IMAR+DEMAR images were performed as previously reported [14] using a custom built Matlab software tool (Version R2011b, MathWorks, Natick, MA). In each patient, a polygon with an individual configuration was drawn around the bone, including the osteosynthetic device and surrounding tissue in five representative slices. Polygons were propagated to all image series (NOMAR, DEMAR, IMAR and IMAR+DEMAR) and attenuation values of successive image pixels on the polygon line were extracted. To quantify density changes on the circle, a discrete Fourier transform of the angle-dependent functions was performed. Resulting spectra show metal artefacts as high amplitudes at low frequencies, whereas image noise is displayed at higher frequencies. Therefore, Fourier coefficients of the lower frequencies (first and second, third and fourth, fifth to eight, and ninth to sixteenth) were analysed and compared between NOMAR, DEMAR, IMAR and IMAR+DEMAR (Fig 2).

Additionally, image noise of NOMAR, DEMAR, IMAR and IMAR+DEMAR was determined in a slice without metal artefacts, by measuring standard deviation in a circular region of interest (ROI) placed in soft tissue.

Reconstruction time of different MAR methods was determined using a microchronometer and restricted to the effective post processing procedure (i.e. excluding data transfer from PACS).

Radiation dose of each examination was estimated by the dose protocol of the scanner.

### Statistical analysis

All statistical analyses were performed using JMP 10.0.0 for Windows (SAS Institute, Cary, NC). Inter-observer agreement of subjective image analysis was determined using Cohen kappa (κ) statistics (values of 0–0.20, 0.21–0.40, 0.41–0.60, 0.61–0.80, and 0.81–1.00 were considered to represent slight, fair, moderate, substantial, and almost perfect agreement, respectively). Normality of the data was tested using Shapiro-Wilk test. Due to non-normally distributed values, comparison of Fourier coefficients and means of subjective analysis from NOMAR, DEMAR, IMAR and IMAR+DEMAR were performed using Mann-Whitney U test and Wilcoxon signed rank test. Results are given as mean ± standard deviation. A p-value less than 0.05 was considered to indicate statistical significance.

## Results

During November and December 2014, a total of 46 patients (women: 50%, mean age: 63±15 years) were included in the analysis (Table 1), 36% had a hip prosthesis (n = 17, in three patients bilateral) and 64% had metallic dental implants (n = 30). Time for reconstruction of IMAR and standard reconstructions was not significantly different, but post-processing time for DEMAR, comparing with IMAR was significantly longer due to separate work-flow (0.6±0.003 vs. 1.6±0.06 seconds per slice; respectively). Given the fact that the initial quantitative and qualitative image evaluation indicated different effects of MAR on hip prosthesis and dental implants, a stratified analysis was pursued.

### Qualitative Image Analysis of MAR

The inter-observer agreement for all MAR methods was high to excellent, with kappa values ranging between 0.73 and 1.00 (Table 2). The disagreement between the two readers was predominantly (75%) due to a difference in judging the impact of artefacts on distant tissue.

**Table 2:** Cohens kappa show interrater reliability of MAR methods stratified by hip prosthesis and dental implants. NOMAR = no metal artefact reduction, DEMAR = Dual-energy metal artefact reduction, IMAR = iterative metal artefact reduction, IMAR+DEMAR = combination of IMAR and DEMAR.

MAR method	Artefact	Tissue adjacent	Tissue distant
**HIP PROSTHESIS**			
** NOMAR**	1.00	1.00	0.91
** DEMAR**	0.75	0.75	0.92
** IMAR**	0.88	0.81	0.90
** IMAR+DEMAR**	0.83	0.77	0.82
**DENTAL IMPLANTS**			
** NOMAR**	1.00	0.82	0.81
** DEMAR**	0.92	0.86	0.76
** IMAR**	0.83	0.75	0.76
** IMAR+DEMAR**	0.81	0.88	0.73

**Fig 3:**
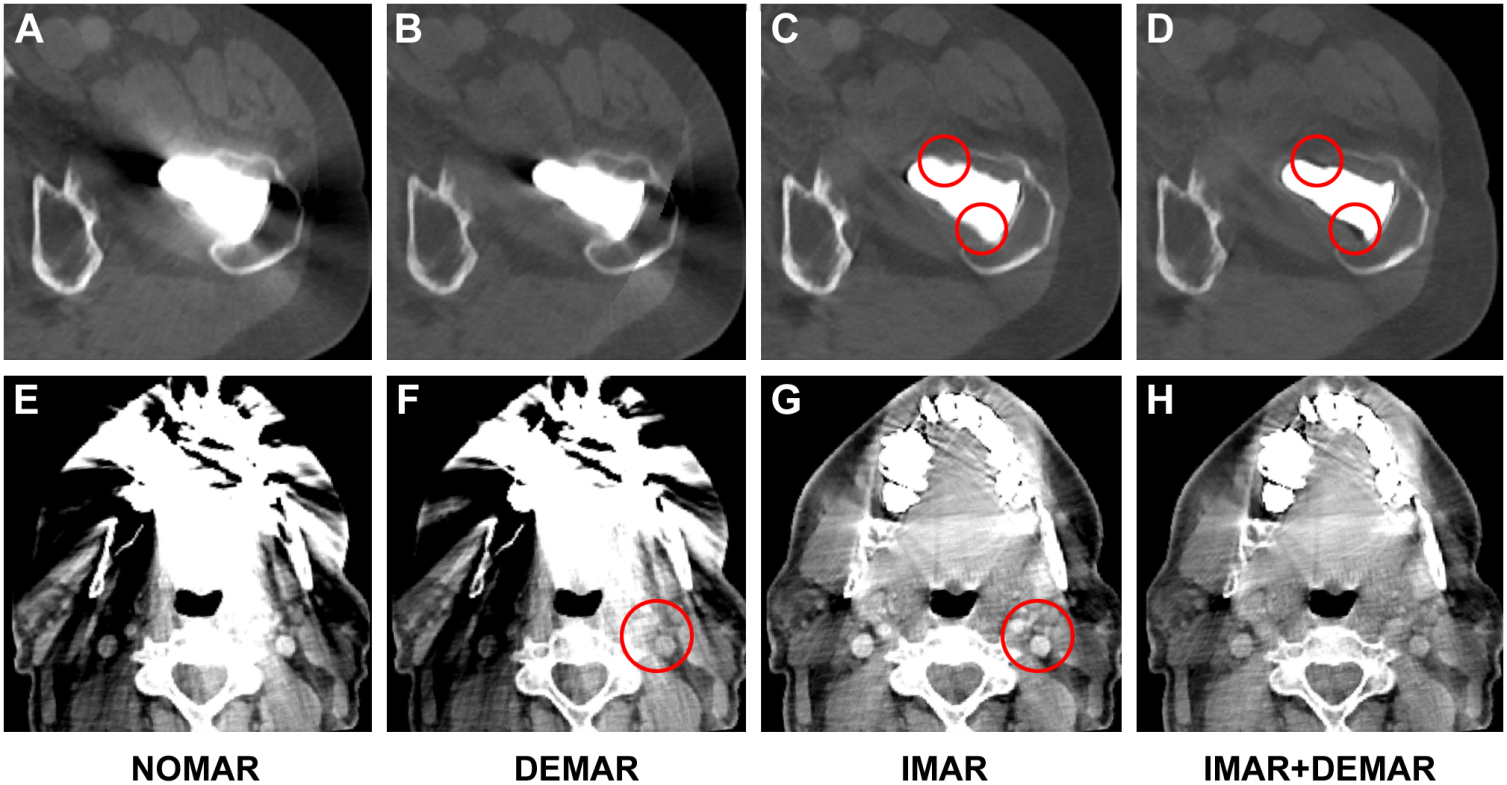
Examples of hip prosthesis in bone window (A-D) and dental implants in abdominal window (E-H). Incremental effect of DEMAR in combination with IMAR inverts remaining high contrast artefacts of IMAR and allows a better evaluation of the prosthesis (red circles in C and D). In terms of contrast enhanced images, DEMAR results in contrast attenuation (red circles in F and G). NOMAR = no metal artefact reduction, DEMAR = Dual-energy metal artefact reduction, IMAR = iterative metal artefact reduction, IMAR+DEMAR = combination of IMAR and DEMAR.

**Fig 4:**
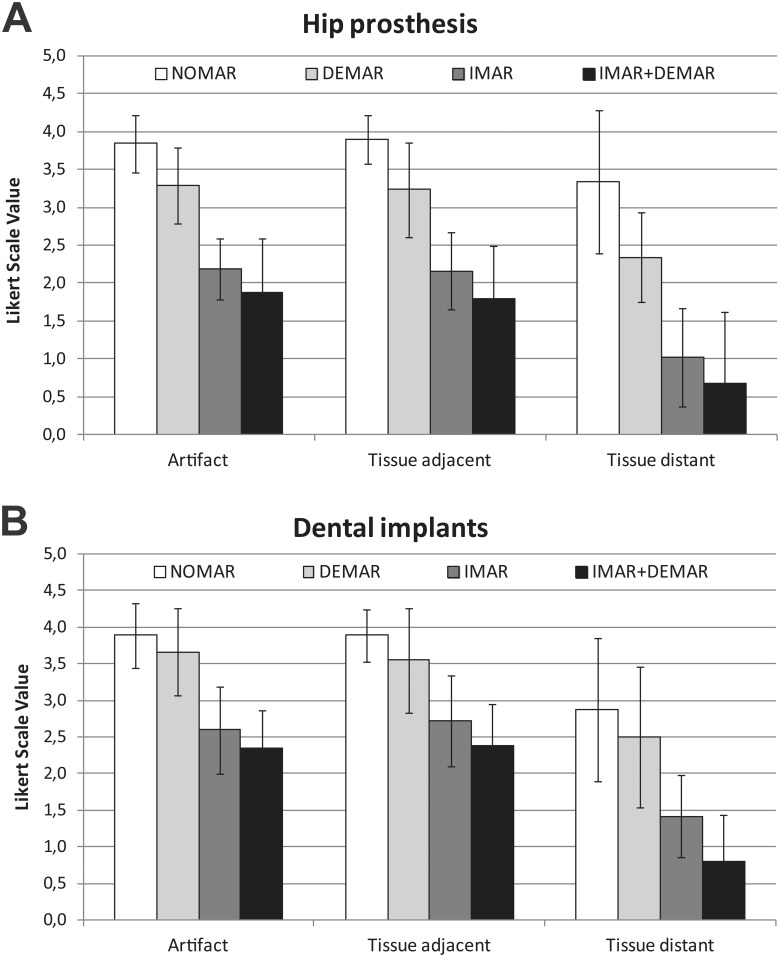
Bar-graphs demonstrating qualitative assessment of impact on metal artefacts of the different MAR approaches applied A) hip prosthesis and B) dental implants based on 5 point Likert scales. NOMAR = no metal artefact reduction, DEMAR = Dual-energy metal artefact reduction, IMAR = iterative metal artefact reduction, IMAR+DEMAR = combination of IMAR and DEMAR.

The results of the qualitative image analysis are shown in Figs 3 and 4, respectively Tables 3 and 4. In subjects with hip prostheses, DEMAR significantly reduces artefacts in comparison to NOMAR from 3.84 ± 0.37 to 3.29 ± 0.71 Likert points (p = 0.0045) with a reduction of artefacts in more distant tissue from 3.34 ± 0.94 to 2.34 ± 0.94 Likert points (p = 0.0018). Also, IMAR resulted in significantly reduced artefacts in comparison to NOMAR to 2.18 ± 0.51 Likert points (p<0.0001) and also in an increased effect of artefact reduction in distant tissue to 1.03 ± 0.59 Likert points (p<0.0001). In a direct comparison, the effect of IMAR on reducing artefacts of hip prosthesis was significantly higher than of DEMAR (all p <0.005). There was an almost significant qualitative difference between DEMAR in combination with IMAR as compared with IMAR alone (p = 0.052).

**Table 3:** Comparison of the means of qualitative (Likert scale) and quantitative (fourier coefficients) image evaluation of NOMAR, DEMAR, IMAR and IMAR+DEMAR at hip prosthesis. NOMAR = no metal artifact reduction, DEMAR = Dual-energy metal artifact reduction, IMAR = iterative metal artifact reduction, IMAR+DEMAR = combination of IMAR and DEMAR, p = p value, n.s. = non-significant.

	NOMAR	DEMAR	p*	IMAR	p*	IMAR+DEMAR	p*	p**	p***
**Qualitative analysis**									
**Artifact**	3.84 ± 0.37	3.29 ± 0.71	0.0045	2.18 ± 0.51	<0.0001	1.87 ± 0.40	<0.0001	<0.0001	0.0518
**Tissue adjacent**	3.89 ± 0.32	3.24 ± 0.69	0.0005	2.16 ± 0.62	<0.0001	1.79 ± 0.51	<0.0001	<0.0001	0.0559
**Tissue distant**	3.34 ± 0.94	2.34 ± 0.94	0.0018	1.03 ± 0.59	<0.0001	0.68 ± 0.65	<0.0001	0.0001	0.0709
**Quantitative analysis**									
**Streaks**	137035 ± 101765	91991 ± 96934	<0.0001	60558 ± 43022	<0.0001	32359 ± 18567	<0.0001	0.0015	<0.0001
		(-32.87%)		(-55.81%)		(-76.39%)		(-33.17%)	(-46.57%)

* Comparison of the means vs. NOMAR

**Comparison of the means IMAR vs. DEMAR.

*** Comparison of the means IMAR vs. IMAR+DEMAR

**Table 4:** Comparison of the means of qualitative (Likert scale) and quantitative (fourier coefficients) image evaluation of NOMAR, DEMAR, IMAR and IMAR+DEMAR at dental implants. NOMAR = no metal artifact reduction, DEMAR = Dual-energy metal artifact reduction, IMAR = iterative metal artifact reduction, IMAR+DEMAR = combination of IMAR and DEMAR, p = p value, n.s. = non-significant.

	NOMAR	DEMAR	p*	IMAR	p*	IMAR+DEMAR	p*	p**	p***
Qualitative analysis									
Artifact	3.89 ± 0.44	3.66 ± 0.60	0.0309	2.60 ± 0.60	<0.0001	2.33 ± 0.52	<0.0001	<0.0001	0.0880
Tissue adjacent	3.89 ± 0.36	3.54 ± 0.78	0.0301	2.73 ± 0.46	<0.0001	2.39 ± 0.57	<0.0001	<0.0001	0.0158
Tissue distant	2.87 ± 0.97	2.50 ± 0.96	0.0892	1.42 ± 0.56	<0.0001	0.81 ± 0.63	<0.0001	0.0001	0.0004
Quantitative analysis									
Streaks	255720 ± 148377	234076 ± 150932	0.106	73877 ± 37441	<0.0001	60455 ± 29116	<0.0001	0.0014	<0.0001
		(-8.46%)		(-71.11%)		(-76.36%)		(-68.44%)	(-18.17%)

* Comparison of the means vs. NOMAR

**Comparison of the means IMAR vs. DEMAR.

*** Comparison of the means IMAR vs. IMAR+DEMAR

In subjects with dental implants, DEMAR subjectively reduced artefacts in comparison to NOMAR from 3.89 ± 0.44 to 3.66 ± 0.60 Likert points (p = 0.03), with an artefact reduction in distant tissue from 2.87 ± 0.97 to 2.5 ± 0.96 Likert points (p = 0.089). Also, IMAR resulted in significantly lowered artefacts in comparison to NOMAR with 2.6 ± 0.60 Likert points (p<0.0001) and an even better effect in distant tissue (1.42 ± 0.56 Likert points, p<0.0001). Again, in the direct comparison, the effect of IMAR on reducing artefacts of dental implants was significantly higher than from DEMAR (all p<0.0001). Combination of DEMAR and IMAR resulted in higher artefact reduction than IMAR, particularly in distant tissue (adjacent p = 0.088 and on distant tissue p <0.005).

### Quantitative Image Analysis of MAR

**Fig 5:**
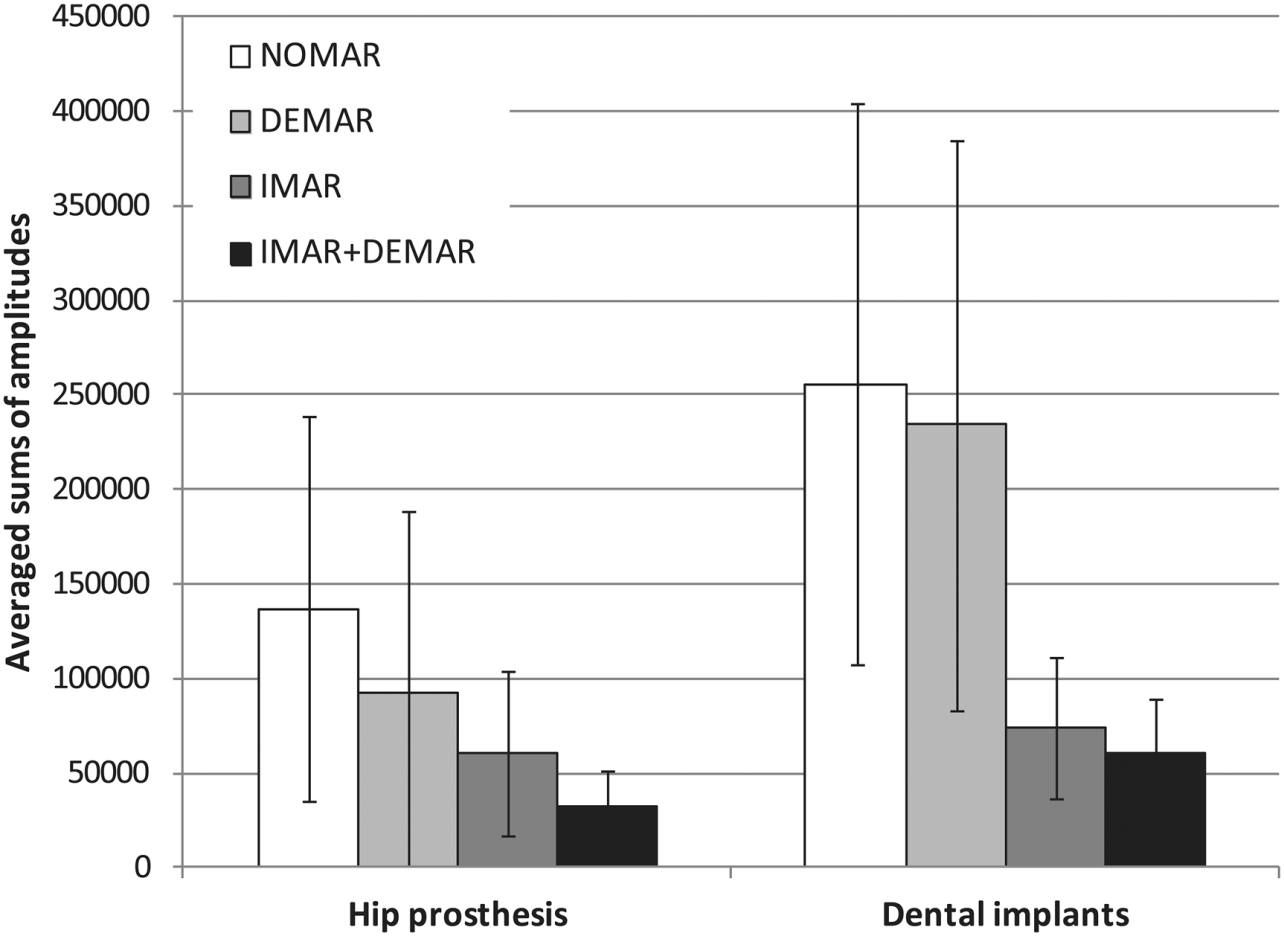
Bar-graphs demonstrating the association between the different MAR approaches applied and quantitative reduction of metal artefacts (averaged sums of amplitudes of the lower frequencies) representing streaking artefacts from hip prosthesis and metallic dental implants. NOMAR = no metal artefact reduction, DEMAR = dual-energy metal artefact reduction, IMAR = iterative metal artefact reduction, IMAR+DEMAR = sequential combination of iterative and dual-energy metal artefact reduction.

Results of quantitative image analysis are shown in Fig 5 and Tables 3 and 4.

Comparing amplitudes of Fourier coefficients from hip prosthesis, DEMAR and IMAR showed significant lower artefact values than NOMAR (all p<0.0001), while IMAR showed significant lower values than DEMAR (p = 0.0015). The combination of IMAR and DEMAR resulted in significantly lower artefact values as compared with IMAR alone (p<0.0001).

In subjects with dental implants, there was no significant difference between DEMAR and NOMAR, however, IMAR resulted in significantly lower values as compared with NOMAR and DEMAR (all p = <0.0001).

The combination of IMAR and DEMAR resulted in a highly significant artefact reduction than IMAR alone (p<0.0001).

## Discussion

In the present study we systematically evaluated the effect of different approaches of metal artefact reduction for dental implants and hip prosthesis in oncological patients undergoing routine contrast enhanced whole body CT follow up. Our results indicate that both approaches evaluated, DEMAR and IMAR, resulted in significant reduction of metal artefacts and allow for improved diagnostic assessment of the implant, the surrounding tissue as well as the interface between implant and adjacent tissue. The results demonstrate that IMAR provides higher reduction of metal artefacts, when compared to DEMAR. Moreover, combining IMAR and DEMAR leads to an incremental benefit compared to the single methods.

Currently, two major approaches have been translated into a clinical routine [15]. While monoenergetic extrapolation of higher kV-values is based on dual-energy acquisitions [6, 16 – 18], iterative reconstruction algorithms have been applied, which also showed diagnostically relevant impact [9, 19].

Compared with prior research, we confirm the effect of both DEMAR and IMAR on the degree of metal artefact reduction. In a smaller study of 33 patients, Han et al. depicted the increase of diagnostic confidence in the assessment of the pelvic cavity when applying DEMAR in patients with hip prostheses, but he also described the introduction of new artefacts [5], which were not detected in our study. We also confirm earlier work by Morsbach et al. who showed a significant reduction of artefacts from hip prosthesis by IMAR, resulting in higher reliability of Hounsfield Units and confidence for depicting pelvic abnormalities [7]. However, in contrast to prior research, we directly compared the two approaches, which may be of particular relevance to a clinical applicability of these techniques, given the limited availability of resources to apply different methods simultaneously. As such, our findings indicate that DEMAR needs more reconstruction time and that IMAR may provide highest reduction of metal artefacts when used as a single application. One explanation may be that DEMAR imitates higher tube voltages, without compensation of remaining artefacts. On the contrary, IMAR discards projections provoking artefacts and interpolates missing information from distant projections. It is important to note that by using, IMAR the visualization of contrast enhancement from intravenous administration of contrast material remained unaffected as compared to DEMAR. Due to the low iodine k-edge of 33.2 keV, monoenergetic extrapolations at high-energy, as used for DEMAR, reduce soft tissue contrast especially after intravenous contrast administration [11].

Our results revealed a significant benefit by combining IMAR and DEMAR in quantitative analysis. In qualitative analysis statistical significance could be shown at dental implants in adjacent and distant tissue, but only nearly reached at hip prosthesis.

The other major finding of our study is that the combination of IMAR and DEMAR results in further additional reduction of artefacts, but the comparison did not reach complete statistical significance in the qualitative analysis, which may be attributed to a limited sample size and small effect size. One explanation for this finding may be that by combining IMAR with DEMAR, a better evaluation of the structural integrity of the implant itself is feasible, due to inverting residual high contrast artefacts of IMAR adjacent to metal implants. However, the combination of IMAR and DEMAR may hold further potential for quantitative DE-analysis.

There are a number of limitations to this retrospective study. Certainly, we did not specifically determine the impact of DEMAR and IMAR on different implant material, given the lack of information concerning detailed composition of the investigated implants in our cohort. It is known that different alloys and especially the volume of used metals have an impact on the efficacy of MAR methods; thus, more systematic research presumably in a comprehensive ex-vivo setting is warranted. Also, we did not specifically study the impact on the assessment of distinct pathologies (i.e. oral cavity carcinomas or pelvic malignancies) routinely covered by metal artefacts, but rather relied on measurements in healthy reference tissue. Thus, more subgroup-specific analyses would be desirable. While we did not assess the superiority of the reduction of MAR between qualitative or quantitative measures, both findings assess the differences between a radiological objective evaluation and measurable differences but need to be evaluated within the same context. Finally, we did not apply a gold standard for artefact-free tissue, which would rather suited in an ex-vivo study set-up.

### Conclusions

In conclusion, IMAR allows for higher reduction of metal artefacts caused by hip prostheses and dental implants, compared to a dual energy based method. The combination of DE-source images with IMAR and subsequent monoenergetic extrapolation provides an incremental benefit compared to both single methods.

## Supporting Information

S1 Dataset Results of subjective image analysis. (XLSX)

S2 Dataset Results of objective image analysis. (XLSX)
